# Heritability and circulating concentrations of pregnancy-associated plasma protein-A and stanniocalcin-2 in elderly monozygotic and dizygotic twins

**DOI:** 10.3389/fendo.2023.1193742

**Published:** 2023-06-02

**Authors:** Rikke Hjortebjerg, Dorthe Almind Pedersen, Jonas Mengel-From, Louise Helskov Jørgensen, Kaare Christensen, Jan Frystyk

**Affiliations:** ^1^ Endocrine Research Unit, Department of Endocrinology, Odense University Hospital, Odense, Denmark; ^2^ Department of Clinical Research, Faculty of Health Sciences, University of Southern Denmark, Odense, Denmark; ^3^ Steno Diabetes Center Odense, Odense University Hospital, Odense, Denmark; ^4^ The Danish Twin Registry and Danish Aging Research Center, University of Southern Denmark, Odense, Denmark; ^5^ Department of Clinical Genetics, Odense University Hospital, Odense, Denmark; ^6^ Department of Biochemistry, Odense University Hospital, Odense, Denmark

**Keywords:** aging, IGF-I, IGF-II, PAPP-A, STC2, twin

## Abstract

**Introduction:**

Pregnancy-associated plasma protein-A (PAPP-A) is an IGF-activating enzyme suggested to influence aging-related diseases. However, knowledge on serum PAPP-A concentration and regulation in elderly subjects is limited. Therefore, we measured serum PAPP-A in elderly same-sex monozygotic (MZ) and dizygotic (DZ) twins, as this allowed us to describe the age-relationship of PAPP-A, and to test the hypothesis that serum PAPP-A concentrations are genetically determined. As PAPP-A is functionally related to stanniocalcin-2 (STC2), an endogenous PAPP-A inhibitor, we included measurements on STC2 as well as IGF-I and IGF-II.

**Methods:**

The twin cohort contained 596 subjects (250 MZ twins, 346 DZ twins), whereof 33% were males. The age ranged from 73.2 to 94.3 (mean 78.8) years. Serum was analyzed for PAPP-A, STC2, IGF-I, and IGF-II by commercial immunoassays.

**Results:**

In the twin cohort, PAPP-A increased with age (r=0.19; *P<*0.05), whereas IGF-I decreased (r=-0.12; *P<*0.05). Neither STC2 nor IGF-II showed any age relationship. When analyzed according to sex, PAPP-A correlated positively with age in males (r=0.18; *P<*0.05) and females (r=0.25; *P<*0.01), whereas IGF-I correlated inversely in females only (r=-0.15; *P<*0.01). Males had higher levels of PAPP-A (29%), STC2 (18%) and IGF-I (19%), whereas serum IGF-II was 28% higher in females (all *P<*0.001). For all four proteins, within-pair correlations were significantly higher for MZ twins than for DZ twins, and they demonstrated substantial and significant heritability, which after adjustment for age and sex averaged 59% for PAPP-A, 66% for STC2, 58% for IGF-I, and 52% for IGF-II.

**Discussion:**

This twin study confirms our hypothesis that the heritability of PAPP-A serum concentrations is substantial, and the same is true for STC2. As regards the age relationship, PAPP-A increases with age, whereas STC2 remains unchanged, thereby supporting the idea that the ability of STC2 to inhibit PAPP-A enzymatic activity decreases with increasing age.

## Introduction

Pregnancy-associated plasma protein-A (PAPP-A) and stanniocalcin-2 (STC2) are physiologically related proteins that are involved in insulin-like growth factor (IGF) signaling and pathologies relevant to human aging. STC2 is a secreted protein that is widely expressed in various tissues, including a broad spectrum of tumors, where its presence has been positively correlated with tumor growth, invasion, metastasis, and patient outcome (1, 2). The deleterious effect of STC2 on cancer survival may relate to its anti-apoptotic and pro-angiogenetic effects combined with its ability to protect tumor cells against hypoxia and oxidative stress (2). In addition, recent clinical studies suggest a link between STC2 and glucose metabolism. Thus, circulating STC2 decreased in parallel with improvements in the glucose regulation that accompanies bariatric surgery (3) and metformin treatment (4). Finally, STC2 has recently emerged as a regulator of PAPP-A (5), which appears to be linked to aging (6).

In the circulation as well as in tissue fluids, the vast majority of IGF-I is bound to IGF-binding proteins (IGFBPs) that inhibits IGF-I from interaction with its target, the IGF-I receptor (7). Thus, to be able to activate its receptor, IGF-I needs to be liberated from the IGFBPs. This liberation is controlled by proteolytic enzymes, e.g. PAPP-A. This enzyme is present in the circulation, but it is in tissue fluids and at cell surfaces that PAPP-A exerts its action: to cleave IGFBPs into fragments with low IGF-I affinity, hereby increasing local IGF-I action (7–10). PAPP-A does not cleave all six IGFBPs, but only IGFBP-2, -4 and -5. However, in contrast to IGFBP-2 and IGFBP-5, no other proteolytic enzymes are known to target IGFBP-4 under physiological conditions (11) and consequently, IGFBP-4 is believed to be the principal substrate for PAPP-A (9).

IGFBP-4 constitute about 10% of the combined pool of circulating IGFBPs, and consequently, IGFBP-4 carries only a small fraction of the circulating IGFs (12). This implies that in humans the physiological role of PAPP-A is to fine-tune tissue IGF-I action. However, studies in mice show that in the extreme situation, when PAPP-A is genetically knocked out PAPP-A may have major effects on body growth (12).

STC2 was recently discovered as an inhibitor of PAPP-A enzymatic activity through formation of covalent complexes with PAPP-A, whereby PAPP-A becomes unable to liberate IGF-I (5). Thus, STC2 may be considered as an inhibitor of IGF-I action. The physiological link between PAPP-A, STC2 and IGF-I has been well documented in rodents. Genetic overexpression of STC2 produces dwarf mice (13) and this effect has been specifically linked to a STC2-mediated inhibition of PAPP-A enzymatic activity (5). Besides controlling body growth, preclinical studies have demonstrated that PAPP-A is pro-atherosclerotic, pro-tumorigenic and furthermore, involved in processes controlling health- and life-span (11, 14–16). In mice, genetic silencing of PAPP-A prolongs the life-span by 20-30% (17, 18), and this observation has fueled the concept that inhibition of PAPP-A enzymatic activity constitutes an attractive target for anti-aging and age-related diseases (6).

Despite the potential roles of PAPP-A and STC2 in aging-related diseases, there is little to no information on their serum concentrations and regulation in elderly people. Therefore, we found it of interest to describe PAPP-A and STC2 serum concentrations in elderly subjects, and to test the hypothesis that a large proportion of their serum concentration variability can be ascribed to genetic factors. To generate such data, we capitalized on our access to a very well characterized cohort of Danish dizygotic (DZ) and monozygotic (MZ) twins (19). To evaluate PAPP-A and STC2 in an IGF context, we also studied the heritability and age-dependency of IGF-I and IGF-II (20–25).

## Materials and methods

### Subjects

The serum samples used in this study originated from the repository of the Longitudinal Study of Aging Danish Twins (LSADT), which was a cohort-sequential study initiated in 1995 with follow-up every second year until 2005 (19, 26). During a LSADT survey in 1997, 689 twins aged +73 years provided blood samples. From the original sample, we had access to serum from 596 subjects from 298 same-sex twin pairs, of whom 194 subjects were males (33%) and 402 females (67%). The mean age of the cohort was 78.8 years (ranging from 73.2 to 94.3 years). Of the twins, 250 were MZ twins from 125 intact pairs and 346 were DZ twins from 173 intact pairs. Determination of zygosity is questionnaire-based with four questions about the degree of similarity between the co-twins. Zygosity has been validated using either serological or genetic markers in a subgroup of the twins and the misclassification rate has been found to be less than 5% (27). The project was approved by the Local Scientific Ethical Committee of The Region of Southern Denmark.

### Assays

Serum concentrations of IGF-I, IGF-II, PAPP-A, and STC2 were measured at the Department of Biochemistry, Odense University Hospital, Denmark. None of the serum samples had been thawed prior to the analyses performed in this study, and they had been stored at -80 degrees Celsius since the day of collection.

Serum IGF-I was measured by an IDS-iSYS multi-discipline automated analyzer (Immunodiagnostic Systems Nordic SA, Denmark), as previously published (25). The IGF-I assay has an assay-to-assay coefficient of variation (CV) ranging from 3.4% to 8.7%, and a within-assay CV from 1.3% to 3.7% (25). Serum concentrations of IGF-II, PAPP-A, and STC2 were determined by commercial ELISA kits from AnshLabs (Webster, Texas, USA). Due to sample volume limitations, all ELISA samples were analyzed as single determinations, using a fully automated Triturus immunoassay analyzer (Grifols, Spain). Eight plates were sufficient to measure all samples. Plate-to-plate variation was determined by two internal controls (low and high), which were included on all plates. The plate-to-plate CVs averaged (low and high) 5.3% and 2.7% for IGF-II, 3.6% and 4.5% for STC2 and 1.1% and 2.6% for PAPP-A. According to the manufacturer of the assays, the within-assay CV was <5% for IGF-II and PAPP-A. For STC2, the with-in assay CV was 10.4% at a level of 4.42 ng/mL, decreasing to <5.3% for STC2 levels at 14.70 ng/mL and above. For comparison, the STC2 levels that we measured ranged between 9.9 and 77.2 ng/mL.

### Statistics

Potential correlation within twin pairs (twin dependency) may underestimate the standard errors and was adjusted for in the analyses using each pair as a clustering unit. Thus, the significance level of Pearson correlation coefficients between the four serum concentrations and between each serum concentration and age were obtained using the cluster-option in Stata on twin pairs. The cluster-option automatically induces the use of robust standard error estimation to account for correlation between observations within twin pairs.

MZ twins share all their genes, whereas DZ twins on average share half of their genetic. Therefore, greater similarity in MZ twins than in DZ twins implies the existence of a genetic component in the etiology of the investigated phenotype. We assessed the similarity of MZ and DZ twins calculating Pearson correlations and Intraclass Correlation Coefficient (ICC) for serum concentrations of each of the four proteins for MZ and DZ twins, respectively.

Standard biometrical heritability analyses were performed to estimate the relative contribution of genetic and environmental factors. According to standard biometric practice, the total phenotypic variance (V) can be divided into four different variance components V = A + D + C + E, where A represents additive genetic effects (average sum of effects of alleles across and within loci), D represents the dominant genetic effects (interaction of alleles within loci), C represents the shared environmental effects, and E represents the non-shared environmental effects (28). The broad-sense heritability describes the proportion of the total phenotypic variance that can be explained by genetic variance. The five models ACE, ADE, AE, CE and E was fitted and evaluated to identify the best fitting and parsimonious model. The nested models were evaluated using the Chi-square goodness of fit test. The Akaike Information Criterion (AIC) was used to identify the best fitting non-nested model (with the lowest AIC value).

For each protein, heritability analyses were performed unadjusted and adjusting for age and sex, and furthermore, sensitivity analyses were performed excluding outliers and for transformed data (achieving normal distribution).

The level of statistical significance was set to 0.05. Pearson correlation coefficients (95% CI) and graphics were retrieved using Stata version 17.0 and computation of ICC (95% CI) and heritability analyses were performed using R version 4.1.0 with Mets package version 1.2.9 (29).

## Results

### Inter-individual variance

The serum concentrations of the four proteins all showed a non-Gaussian distribution (*P<*0.05), and accordingly, concentrations are given as median, inter-quartiles, and range (min and max) (**Table 1**). The concentration distributions of the four variables are illustrated in **Supplementary Figure 1**.

**Table 1:** The concentrations of PAPP-A, STC2, IGF-I, and IGF-II in the cohort composed of 596 monozygotic and dizygotic twins.

Variable	min	P25	median	P75	max	max: min ratio	mean (SD)
PAPP-A (ng/mL)	0.30	0.92	1.14	1.43	4.36	14.5	1.21 (0.43)
STC2 (ng/mL)	9.9	24.8	29.3	34.6	77.2	7.8	30.0 (7.8)
IGF-I (ng/mL)	25	71	88	107	283	11.3	92 (32)
IGF-II (ng/mL)	106	328	406	500	903	8.5	413 (126)

n, number; min, minimum; max, maximum; P25, the 25^th^ percentile; P75, the 75^th^ percentile; SD, standard deviation. For none of the four proteins, the concentration distribution complied with the normal distribution (P<0.05). The number of samples was 596 for all four proteins.

### Sex and age differences

**Figure 1:**
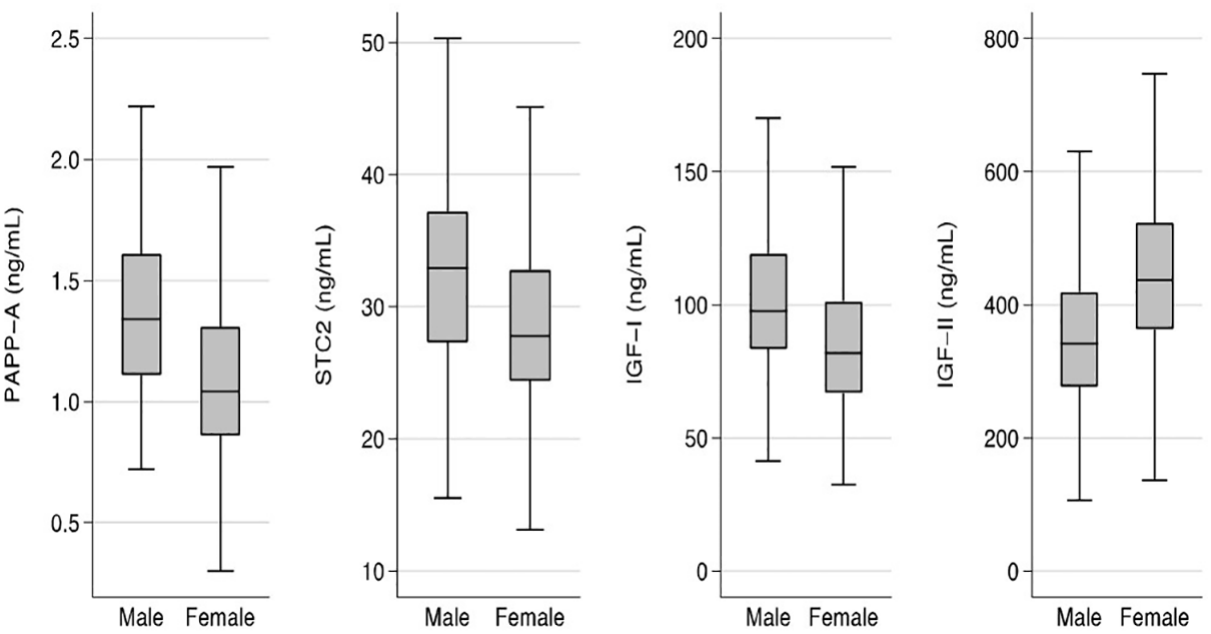
Sex specific concentrations of PAPP-A, STC2, IGF-I, and IGF-II. Box plots of concentrations of the four proteins in males and females. Box represents median with 25th and 75th percentiles and whiskers represent the smallest and largest values that lie within 1.5 times the interquartile range away from the nearer quartile. The differences in concentrations between males and females were significant for all proteins (*P<*0.001). IGF, insulin-like growth factor; PAPP-A, pregnancy-associated plasma protein-A; STC2, stanniocalcin-2.

For all four proteins, the concentrations showed significant sex-differences (**Figure 1**). Median concentrations were higher in males by 29% for PAPP-A, 18% for STC2, and 19% for IGF-I when compared with females, whereas the median concentration of IGF-II was 28% higher in females (all *P-values <*0.001).

**Figure 2:**
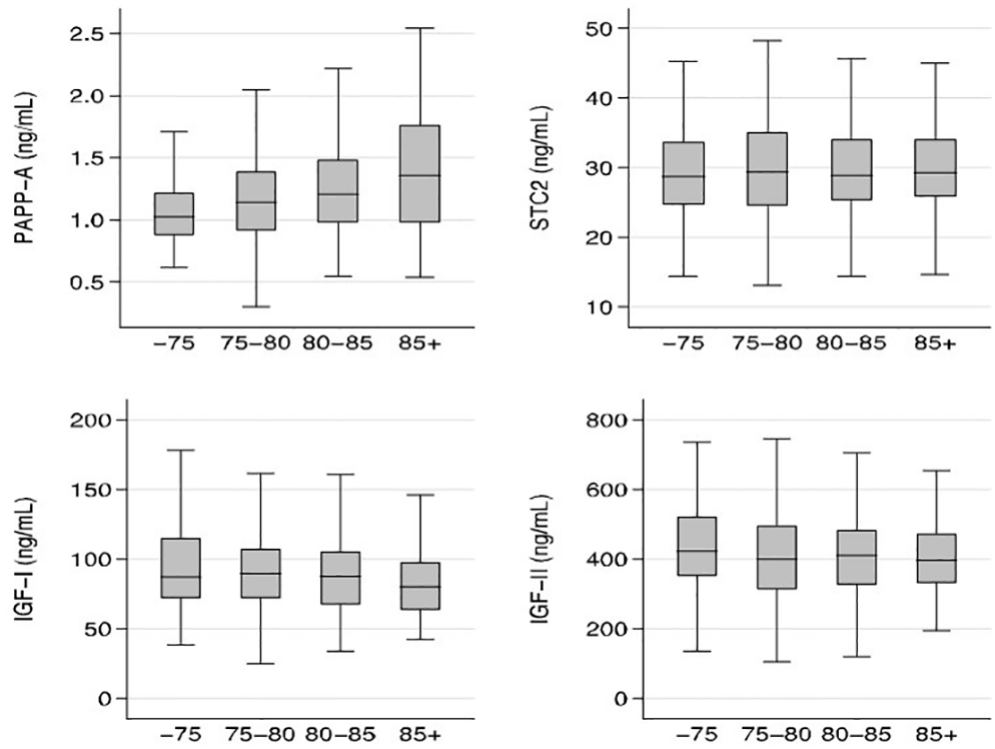
Relationship between age and PAPP-A, STC2, IGF-I, and IGF-II. Box plots of concentrations of the four proteins illustrated according to the following age groups: age ≤75, 75< age ≤80; 80< age ≤85; and >85 years. Box represents median with 25th and 75th percentiles and whiskers represent the smallest and largest values that lie within 1.5 times the interquartile range away from the nearer quartile. There were significant age-relationships for PAPP-A and IGF-I (*P<*0.05) IGF, insulin-like growth factor; PAPP-A, pregnancy-associated plasma protein-A; STC2, stanniocalcin-2.

In the entire cohort, serum PAPP-A increased with age (r=0.19; *P<*0.05), whereas serum IGF-I decreased with age (r=-0.12; *P<*0.05). In contrast, neither STC2 nor IGF-II showed any significant age-related changes. Age relationships of the four proteins are illustrated in **Figure 2**, which shows median concentrations according to the following age groups: age ≤75; 75< age ≤80; 80< age ≤85; and >85 years.

When analyzing the age relationship in males and females separately, some sex differences became apparent. Whereas PAPP-A correlated positively with age in both males (r=0.18; *P<*0.05) and females (r=0.25; *P<*0.01), IGF-I correlated inversely with age in females (r=-0.15; *P<*0.01), but not in males (r=-0.01; *P=*NS).

### Correlations between the four proteins

The strongest correlation observed was between the two IGFs (r=0.32), which both correlated negatively with PAPP-A (IGF-I: r=-0.11; IGF-II: r=-0.19), but positively with STC2 (IGF-I: r=0.25; IGF-II: r=0.10). The weakest correlation was between PAPP-A and STC2 (r=0.09) (all *P-values <*0.05).

### Within-pair correlations and heritability of the four proteins

**Figure 3:**
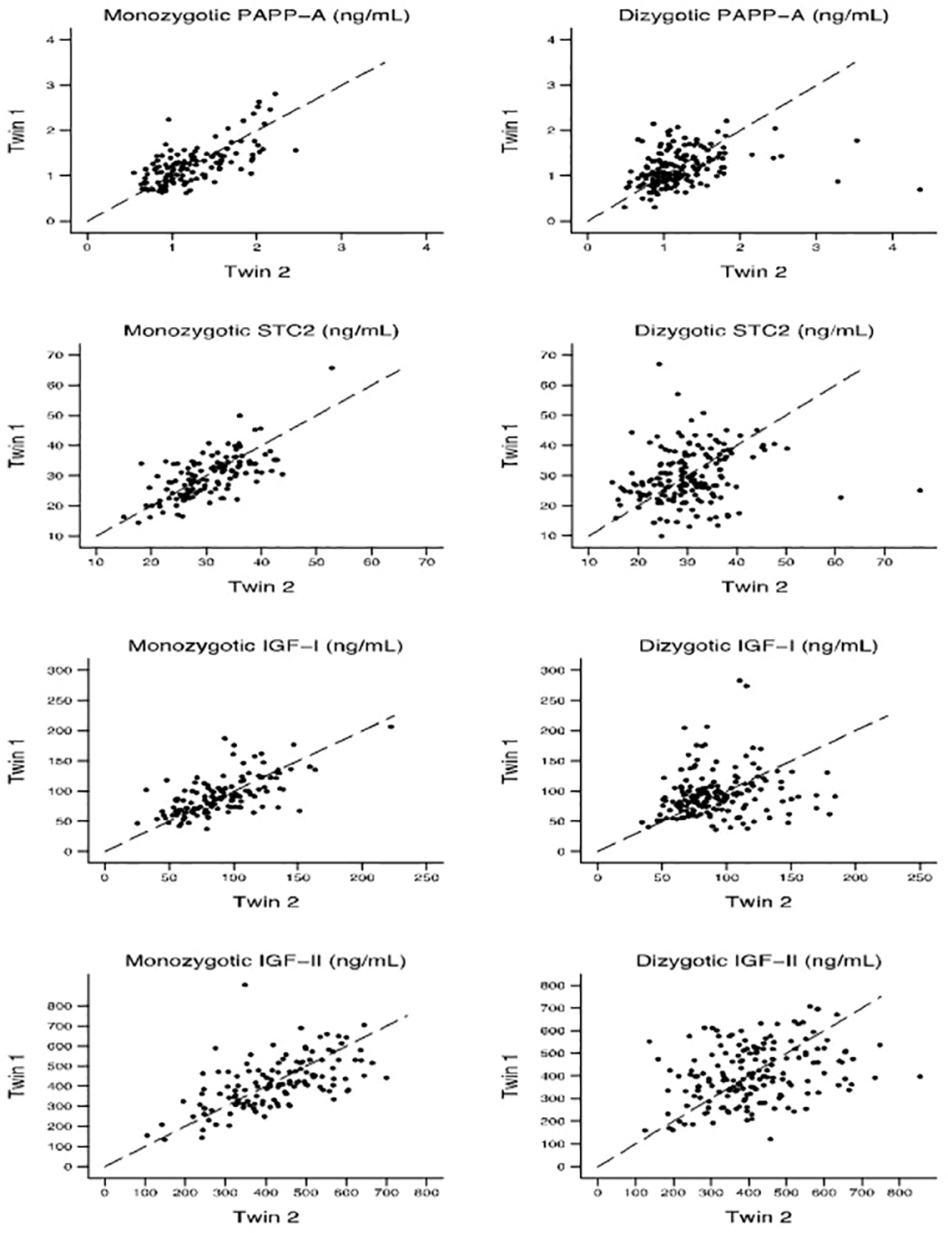
Within-pair resemblance in monozygotic and dizygotic twin pairs for PAPP-A, STC2, IGF-I, and IGF-II. IGF, insulin-like growth factor; PAPP-A, pregnancy-associated plasma protein-A; STC2, stanniocalcin-2.

**Table 2** displays the Pearson correlation coefficients and the ICC (unadjusted and adjusted for age and sex) for each protein serum concentration for MZ and DZ twins, respectively. **Figure 3** shows within-pair resemblance in MZ and DZ twin pairs for PAPP-A, STC2, IGF-I, and IGF-II. Since all were same-sex twins, within-pair variations were not influenced by sex. For all four proteins, the within-pair correlations were significantly stronger for MZ twins than for DZ twins. Excluding or truncating outliers (defied as PAPP-A >3 ng/mL, STC2 >55 ng/mL, IGF-I >200 ng/mL and IGF-I >800 ng/mL) did not change the correlation estimates noteworthy (see **Supplementary Table S1**).

**Table 2:** Within-pair correlations of the serum concentration of PAPP-A, STC2, IGF-I, and IGF-II by Twin Zygosity.

	Twin Zygosity	Pearson correlation [95%CI]	ICC from unadjusted model [95%CI]	ICC from adjusted model* [95%CI]
**PAPP-A**	MZ	0.67 [0.56,0.76]	0.68 [0.59,0.76]	0.63 [0.52,0.72]
	DZ	0.28 [0.14,0.41]	0.26 [0.12,0.39]	0.16 [0.02,0.30]
**STC2**	MZ	0.68 [0.57,0.76]	0.74 [0.66,0.80]	0.72 [0.63,0.79]
	DZ	0.20 [0.05,0.34]	0.18 [0.04,0.30]	0.13 [-0.004,0.26]
**IGF-I**	MZ	0.64 [0.52,0.73]	0.66 [0.56,0.74]	0.64 [0.53,0.73]
	DZ	0.16 [0.01,0.31]	0.15 [0.01,0.28]	0.08 [-0.06,0.22]
**IGF-II**	MZ	0.58 [0.46,0.69]	0.59 [0.47,0.68]	0.52 [0.38,0.63]
	DZ	0.31 [0.17,0.44]	0.31 [0.18,0.44]	0.26 [0.13,0.39]

CI, confidence interval; DZ, dizygotic; ICC, Intraclass Correlation Coefficient; IGF, insulin-like growth factor; MZ, monozygotic; PAPP-A, pregnancy-associated plasma protein-A; STC2, stanniocalcin-2.

*Adjusted for age and sex.

The heritability, that is, the proportion of total phenotypic variance attributable to genetic effects, was estimated from different models. For the unadjusted model, the AE model provided the best fit to PAPP-A and IGF-II data, whereas the ADE model provided a slightly better fit than AE in the analyses of IGF-I and STC2. However, the combined estimate of the overall genetic component of the variance was virtually the same. Therefore, **Table 3** reports the broad-sense heritability from AE models (unadjusted and adjusted for age and sex) for all four proteins while the supplement material gives the results from the ADE models in the cases where it provided a slightly better fit (**Supplementary Table S2**). For all four proteins, the heritability estimates were statistically significant and ranged from 50% to 75%. All estimates were robust to the deletion of outliers and transformation of data to obtain normal distribution of the values (see **Supplementary Tables S3, S4**).

**Table 3:** Heritability for serum concentration of PAPP-A, STC2, IGF-I, and IGF-II from AE model.

Protein	Unadjusted	Adjusted for age and sex
	Heritability [95%CI]	Heritability [95%CI]
**PAPP-A**	0.67 [0.58,0.76]	0.59 [0.48,0.70]
**STC2**	0.71 [0.61,0.80]	0.66 [0.55,0.77]
**IGF-I**	0.62 [0.51,0.73]	0.58 [0.46,0.70]
**IGF-II**	0.59 [0.49,0.69]	0.52 [0.40,0.64]

CI, confidence interval; IGF, insulin-like growth factor; PAPP-A, pregnancy-associated plasma protein-A; STC2, stanniocalcin-2.

## Discussion

The aim of this study was to estimate the heritability of PAPP-A and STC2 and determine their sex and age related serum concentrations in an elderly population of Danish MZ and DZ twins. This knowledge is relevant as both STC2 and PAPP-A appear to be involved in diseases affecting elderly individuals, e.g. cancer (4, 30, 31) and cardiovascular disease (11, 32, 33). To evaluate findings for PAPP-A and STC2 in an IGF-system context, we included data on IGF-I and IGF-II, which carry out many of the biological effects of the IGF-system. The major novel finding was that the heritability of STC2 and PAPP-A appears to explain at least 50% of the variability in their serum concentrations. Another novel finding was the positive association between serum PAPP-A and age, whereas STC2 was found at similar concentrations in all age groups.

Heritability analyses were performed using standard biometric methods (28). By this approach, both PAPP-A and STC2 showed substatial heritability, which reassuringly remained significant after exclusion/truncation of outliers that may strongly influence the associations. The heritability of PAPP-A was recently estimated to 44% by Ferreira et al., but the authors used a different approach. Circulating PAPP-A was determined by an Olink-based assay technique and the heritability by single nucleotide polymorphism (SNP) analysis using a genome-wide association study (GWAS) design (34). Nevertheless, despite the methodological differences, the two studies agree that circulating concentrations of PAPP-A demonstrate a substantial heritable component. As regards STC2, we believe the present data are the first to show that its circulating levels are to a large extent genetically determined. However, even though both serum concentrations of PAPP-A and STC2 appear to be genetically influenced, and circulate without any large diurnal variation (<25%), their serum concentrations appear to be subject to changes (35). Thus, circulating PAPP-A has been reported to exert short-term changes following the intake of a mixed meal, whereas both proteins increase 30 min post-exercise, but to which extent these short-term changes may translate into long-lasting alterations in serum PAPP-A and STC2 concentrations remain uncertain. As regards long-lasting changes, data obtained before and 12 months after a gastric surgery induced weight loss indicate that at least serum STC2 levels are modifiable, as levels decreased by 17%, whereas PAPP-A remained unchanged (3). Finally, it should be stressed that beside the genetic determination of their serum concentrations, also the *biological activity* of STC2 and PAPP-A appears to be influenced by genetic variation. A rare genetic variant in the STC2 gene, yielding a protein variant with reduced ability to inhibit PAPP-A proteolytic activity and thereby casuing an increased IGF-I action, has been reported to associate with an increased body height (36), whereas a common SNP in the PAPP-A gene has been reported to generate an enzyme with less affinity for the IGFBPs and thereby causing reduced IGF-I activity (37). According to GWAS catalogs, no genetic variations in the PAPP-A or STC2 genes have been significantly associated with level of serum IGF-I, whereas genetic variations in the two IGF-genes were associated.

In the present study of elderly subjects, circulating PAPP-A concentrations were higher in males than females, which agrees with findings by others (35, 38, 39). The increase in PAPP-A concentration with age was evident in both males and females and in agreement with earlier findings in younger and middle-aged adults of both sexes in cohorts consisting of healthy subjects (40), patients with chronic stable angina (41), and T2D (39). When compared to our previous studies using the same PAPP-A ELISA as in the present study, levels have been found to be 0.81±0.26 ng/mL in 50 healthy subjects aged 18-50 years (42), and 0.81±.0.27 ng/mL in 99 overweight, but otherwise healthy subjects aged 58±0 years (39). Thus, our finding of a median PAPP-A level of 1.14 (0.92;1.43) ng/mL in subjects of >73 years of age supports the notion that levels continue to increase throughout adult life. The reason for this finding remains to be clarified, but may reflect aging-related changes in PAPP-A synthesis and/or clearance. As regards PAPP-A synthesis, experimental findings in mice indicate that the expression of PAPP-A during aging changes in a tissue specific manner. In some tissues, the expression of PAPP-A increases, while in others it declines. However, at all ages studied, adipose tissue (in particularly visceral adipose tissue) is the major expression site for PAPP-A (43). Because adiposity often increases with age in humans, it is appealing to hypothesize that the age-related increase in serum PAPP-A originates partly from adipose tissue. However, neither obesity (39) nor the major weight reduction that follows bariatric surgery (3) seems to alter circulating PAPP-A concentrations. Thus, changes in adiposity appear to be an unlikely explanation. In contrast, cell culture studies have shown that pro-inflammatory agents stimulate the expression of PAPP-A *in vitro* (8). In this context, TNF-alpha is of particular interest, as earlier studies in the very same twin cohort as we are studying, have shown that circulating TNF-alpha increases when comparing subjects of 73-79 vs. +80 years of age (44). Thus, an age-related increase in TNF-alpha could potentially explain our findings.

Similar to PAPP-A, serum STC2 was higher in males than females, whereas STC2 as opposed to PAPP-A was constant in all age groups for both sexes. This age-independence is in agreement with findings in patients with hepatocellular carcinoma (45), and middle-aged patients with T2D (46). In contrast, Panagiotou et al. (35) found slightly higher STC2 levels (+8%) in females than males (20.0±0.1 years), and reported a weak inverse correlation between serum STC2 and age in 106 euthyroid patients (age range approximately 30 to 80 years of age) with a history of thyroidectomy for non-malignant disease (47). Thus, there appears to be consensus that in adults, STC2 is *not* increasing with age and probably remains more or less unaffected throughout adult life, at least in old age. Hence, in the context of aging, STC2 acts differently than circulating PAPP-A. Given that STC2 inhibits PAPP-A enzymatic activity, it could be speculated whether an aging-related increase in the ratio between PAPP-A to STC2 leads to unbeneficial health for the elderly individuals. However, this idea requires further studies.

The physiological role of IGF-II in adults remains an enigma. Serum levels vary only little with age and pathologies (48), and this may explain why there are sparse data on serum IGF-II and its genetic components in elderly subjects. Nevertheless, knowledge of the magnitude of the genetic component in the variation of circulating IGF-II may be of interest as accumulating evidence suggest that IGF-II play an important role in cancer and metabolic diseases (48). Our data show that serum IGF-II was higher in woman than men, and less heritable than IGF-I. The observation contrasts with original findings by Harella et al., who found that the heritability of IGF-II was almost twice that of IGF-I (66% vs 38%) (20). However, their study was based on small groups (32 MZ and 47 DZ twins), younger subjects (44-77 years of age) and older IGF-assays that no longer comply with contemporary guidelines (20, 49). As regards the age relationship, previous studies of children, adolescents and middle-aged subjects have shown that serum IGF-II remains stable throughout post-natal life (24, 50, 51), and our data extend these findings to include subjects up to ninety years of age. Whether IGF-II at a later time point in life starts to decline is unknown, but data from Vitale et al. indicate that serum IGF-II is reduced by 45% in centenarians as compared to 70-year-old offspring (52).

We compared the heritability of serum IGF-I to the heritability of PAPP-A, STC2 and IGF-II, and we used serum IGF-I to assess whether our cohort in an IGF-system context was comparable to other cohorts of elderly subjects. The present heritability index of IGF-I was in agreement with a recent study by Jensen et al., who found a heritability index of 65% using the same IGF-I assay in a different cohort of Danish twins (22). As regards the age relationship and sex difference of serum IGF-I concentrations in elderly people, our results agreed with some previous findings, but not all (25, 53–55). However, findings regarding IGF-I in people of older age have some controversies (54) – controversies that we believe relate to differences in how the study cohorts were collected and the technical variation due to use of different IGF-I assays, which as previously demonstrated impacts on serum IGF-I concentrations (56, 57). Overall, however, our IGF-I data were as expected and thus, we believe our cohort have provided data that are valid in the context of the IGF-system.

In conclusion, our findings demonstrate a substantial genetic component in the variation in serum PAPP-A and STC2 concentrations. Furthermore, PAPP-A concentrations are about 28% and STC2 about 18% higher in males than females. Finally, serum PAPP-A continues to increase by age in both sexes, whereas STC2 levels remain stable. Given that STC2 inhibits PAPP-A, the latter findings indicate that the enzymatic activity of PAPP-A may increase by increasing age. However, measurements of intact and PAPP-A degraded IGFBP-4 concentrations would have been required to address this question, and unfortunately, we were restricted from these measurements due to sample volume limitations. Accordingly, it remains to be investigated whether the observed aging related changes in PAPP-A has or has not an impact on circulating IGFBP-4 levels.

## Data availability statement

The datasets presented in this article are not readily available because of GDPR. Requests to access the datasets should be directed to KChristensen@health.sdu.dk.

## Ethics statement

The studies involving human participants were reviewed and approved by The Local Scientific Ethical Committee of The Region of Southern Denmark. The patients/participants provided their written informed consent to participate in this study.

## Author contributions

JF, RH, DP, JM-F, and KC designed the study. LJ performed the measurements of PAPP-A, STC2, IGF-I and IGF-II. RH and JF supervised the experimental data. DP made the statistical analyses. RH and JF made the manuscript draft, and all authors reviewed/edited the draft version. All authors contributed to the article and approved the submitted version.
