# Supplementary material for: Heritability and circulating concentrations of pregnancy-associated plasma protein-A and stanniocalcin-2 in elderly monozygotic and dizygotic twins
Source: Front Endocrinol (Lausanne). 2023 Jun 2;14:1193742. doi: 10.3389/fendo.2023.1193742 (PMC10272750; doi:10.3389/fendo.2023.1193742)
Supplement: Supplementary file 1 [file DataSheet_1.pdf]

Supplementary Figures

Figure S1

The distribution of the individual concentrations of PAPP-A, STC2, IGF-I, and IGF-II. For none of the four proteins, the concentration distribution complied with the normal distribution (P<0.05).

IGF, insulin-like growth factor; PAPP-A, pregnancy-associated plasma protein-A; STC2, stanniocalcin-2.

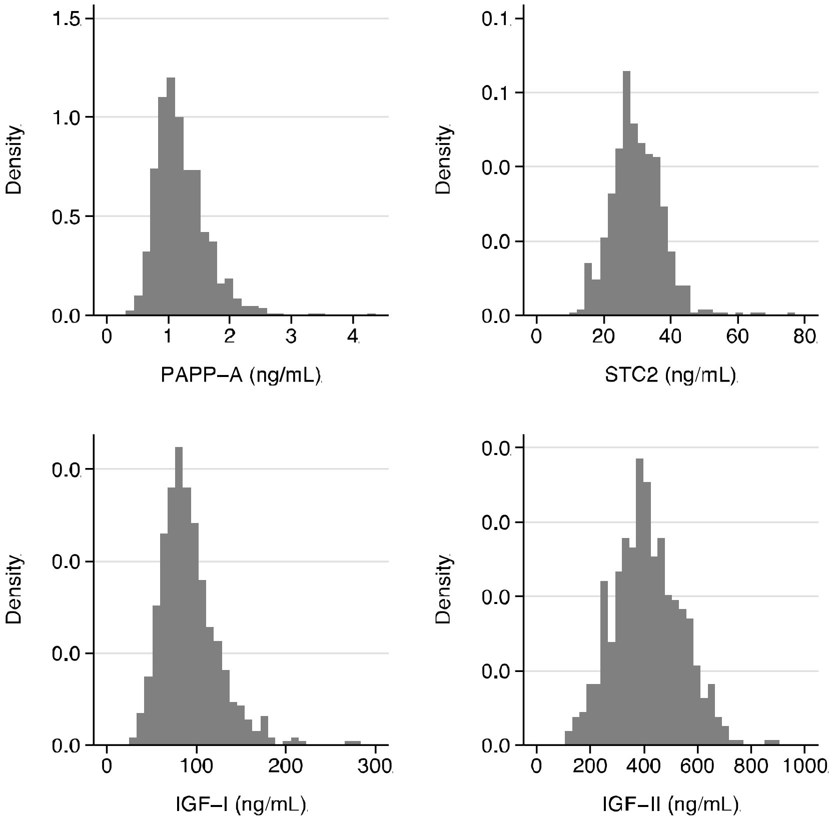

Supplementary Tables

Table S1

Within-pair correlations of the serum concentration of PAPP-A, STC2, IGF-I, and IGF-II excluding and truncating outliers by Twin Zygosity

CI, confidence interval; DZ dizygotic; IGF, insulin-like growth factor; MZ, monozygotic; PAPP-A, pregnancy-associated plasma protein-A; STC2, stanniocalcin-2.

* Outliers were defied as PAPP-A > 3 ng/mL, STC2 > 55 ng/mL, IGF-I > 200 ng/mL and IGF-I > 800 ng/mL

	Twin Zygosity	Number of outliers	Pearson correlation (outliers* excluded)	Pearson correlation (outliers* truncated)
PAPP-A	MZ	0	0.67 [0.56,0.76]	0.67 [0.56,0.76]
	DZ	3	0.42 [0.29,0.54]	0.33 [0.19,0.46]
STC2	MZ	2	0.63 [0.51,0.72]	0.67 [0.56,0.75]
	DZ	4	0.33 [0.19,0.46]	0.24 [0.10,0.38]
IGF-I	MZ	1	0.58 [0.45,0.69]	0.63 [0.51,0.72]
	DZ	1	0.18 [0.03,0.32]	0.16 [0.01,0.30]
IGF-II	MZ	1	0.64 [0.53,0.74]	0.60 [0.48,0.70]
	DZ	4	0.33 [0.19,0.45]	0.32 [0.18,0.44]

Table S2

Heritability for the serum concentration of PAPP-A, STC2, IGF-I, and IGF-II from ADE model in the cases where ADE were a slightly better fit than the AE model

CI, confidence interval; IGF, insulin-like growth factor; PAPP-A, pregnancy-associated plasma protein-A; STC2, stanniocalcin-2.

* AE best fitting model

	Unadjusted	Adjusted for age and sex
	Heritability [95%CI]	Heritability [95%CI]
PAPP-A	*	0.63 [0.53,0.73]
STC2	0.74 [0.67,0.81]	0.71 [0.63,0.80]
IGF-I	0.66 [0.57,0.75]	0.63 [0.53,0.73]
IGF-II	*	*

Table S3

Heritability from the AE model performed on the transformed serum concentration of PAPP-A, STC2, IGF-I, and IGF-II

CI, confidence interval; IGF, insulin-like growth factor; Log(), natural logarithmic transformation; PAPP-A, pregnancy-associated plasma protein-A; sqrt(), square root transformation; STC2, stanniocalcin-2.

* Transformed data for PAPP-A and STC2 deviated slightly from normal distribution, but were closer to normal distribution than non-transformed data.

	Unadjusted	Adjusted for age and sex
	Heritability [95%CI]	Heritability [95%CI]
log(PAPP-A)*	0.66 [0.58,0.75]	0.58 [0.48,0.69]
log(STC2)*	0.71 [0.62,0.80]	0.67 [0.56,0.78]
log(IGF-I)	0.58 [0.48,0.69]	0.55 [0.43,0.66]
sqrt(IGF-II)	0.63 [0.54,0.73]	0.57 [0.46,0.68]

Table S4

Heritability for the serum concentration of PAPP-A, STC2, IGF-I, and IGF-II from the AE model when outliers were excluded

CI, confidence interval; IGF, insulin-like growth factor; PAPP-A, pregnancy-associated plasma protein-A; STC2, stanniocalcin-2.

* Outliers were defied as PAPP-A >3 ng/mL, STC2 >55 ng/mL, IGF-I >200 ng/mL and IGF-I >800 ng/mL

	Unadjusted	Adjusted for age and sex
	Heritability [95%CI]	Heritability [95%CI]
PAPP-A	0.65 [0.57,0.73]	0.58 [0.48,0.67]
STC2	0.68 [0.59,0.77]	0.64 [0.54,0.74]
IGF-I	0.55 [0.44,0.67]	0.52 [0.39,0.64]
IGF-II	0.65 [0.56,0.74]	0.58 [0.48,0.69]
